# Small-scale spontaneous dynamics in temperate beech stands as an importance driver for beetle species richness

**DOI:** 10.1038/s41598-022-16352-7

**Published:** 2022-07-13

**Authors:** Václav Zumr, Jiří Remeš, Oto Nakládal

**Affiliations:** Faculty of Forestry and Wood Sciences, Czech University of Life Sciences Prague, Kamýcká 129, 165 00 Prague 6-Suchdol, Czech Republic

**Keywords:** Ecology, Zoology, Environmental sciences

## Abstract

Natural dynamics in forests play an important role in the lives of many species. In the landscape of managed forests, natural disturbances are reduced by management activities. This usually has a significant effect on insect diversity. The effect of small-scale natural dynamics of protected beech stands on the richness of saproxylic and non-saproxylic beetles was investigated. Sampling was carried out by using flight interception traps in the framework of comparing different developmental stages: optimum, disintegration, and growing up, each utilizing 10 samples. We recorded 290 species in total, of which 61% were saproxylic. The results showed that the highest species richness and thus abundance was in the disintegration stage. In each developmental stage, species variation was explained differently depending on the variable. Deadwood, microhabitats, and canopy openness were the main attributes in the later stages of development for saproxylic beetles. For non-saproxylics, variability was mostly explained by plant cover and canopy openness. Small-scale disturbances, undiminished by management activities, are an important element for biodiversity. They create more structurally diverse stands with a high supply of feeding and living habitats. In forestry practice, these conclusions can be imitated to the creation of small-scale silvicultural systems with active creation or retention of high stumps or lying logs.

## Introduction

Forest ecosystems are one of the most important carriers of regional biodiversity^1^. Over several centuries, forests have been largely changed into managed stands with simple stand structures^2^. Few untouched natural forests have been conserved^3^, usually only in the higher, inaccessible areas^4^. The early stages and final stages of forest development have almost disappeared from the landscape^5^. They are typically caused by natural disturbances^6, 7^.

Invertebrates are strongly affected by anthropogenic landscape changes and are, therefore, in decline globally^8–10^. Beetles are the most studied group of insects, especially saproxylic, deadwood-dependent beetle species^11^, which are also one of the most threatened groups in forests^12, 13^. In fact, for many saproxylic species, the continuity of habitat conditions is more important than the amount of deadwood itself^14^, which is primarily determined by the previous management. Removing all deadwood leads to a decline in saproxylic species^13^. Natural beech forests are of high value for conservation of this group of beetles^15, 16^. Therefore, forest reserves are often newly established in areas where at least an autochthonous tree species composition is observed^17, 18^. The main aim of establishing reserves is to maintain and enhance biodiversity in a specific area^12, 19^. The number of species found in reserves that have been protected for a relatively short period of time is generally lower when compared to ancient protected forests^12^. However, these sites may be a regional refuge for a high number of saproxylic species in the future. In recent years, so-called integrative management, which is characterized by active deadwood enrichment, has been frequently studied^20–22^. If stands around protected areas are managed in this alternative way, species that exclusively inhabit the current reserves may spread to more distant locations^23^.

The reason for protection is also to maintain the natural dynamics of forest stands^24–26^. As a rule, intensive logging has adverse consequences for saproxylic beetle biodiversity when removing wood after a disturbance^7^. The natural development of forests results in the gradual disturbance of homogeneous structures, usually by wind^27, 28^, but also by insect outbreaks and fire^29^. Forests were also shaped by large herbivores in the past^30, 31^. These disturbances resulted in a highly structured heterogeneous landscape^32^. Naturally formed forest stands contain numerous attributes that support biodiversity. Typically, these are high volume and large deadwood dimensions^33^, which are required for the most endangered saproxylic beetle species^34, 35^. Other important attributes for saproxylic beetles are microhabitats^36, 37^ and, due to canopy openness, increased exposure of the interior of stands^38, 39^.

The research focused on the response of beetle (Coleoptera) richness to the natural small-scale dynamics of recently protected beech stands. These dynamics periodically appear in climax forests^40^. Disturbance typically takes place over areas of several ares through the death of a few trees in the group^41, 42^. However, scientific work has focused mostly on large-scale disturbances such as windstorms^43–45^. For this study, three basic derived growth stages of small-scale natural forest development were used according to Korpeľ^46^ and Emborg^47^. Two research questions were posed: (1) Does species richness differ between developmental stages? (2) What variables of the stand in each stage best explain species preference?

## Materials and methods

### Study location

**Figure 1:**
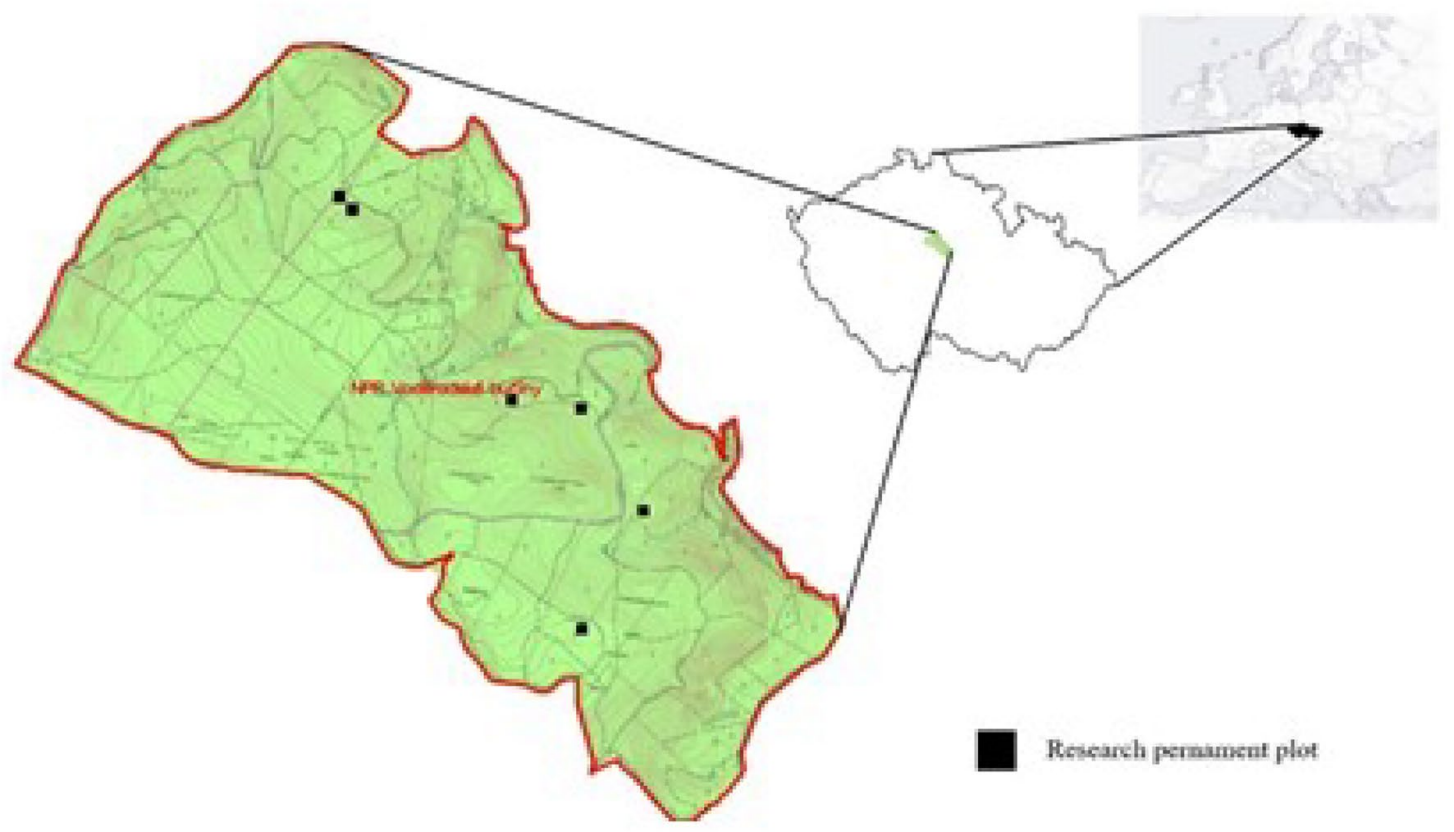
Location of the study area and adjustment of permanent plots in the study area. The map was created in the Paint 3D application (Microsoft, Win10).

The study was carried out in Czech Republic (Central Europe) in the area of the Voděradské bučiny (49° 58′ N, 14° 48′ E). The area is located 40 km east of Prague (Fig. 1). The Voděradské bučiny has an area of 682 ha, dominated by beech (*Fagus sylvatica* L.) stands 180 + years old. The present beech stands were renewed between 1800 and 1820 by natural regeneration through the shelterwood silvicultural system. Predominant forest types are *Fagetum acidophilum* and *Querceto-Fagetum acidophilum*. The completely dominant soil types are Cambisols: modal oligotrophic, dystric, and arenic. The average annual temperature is 7.8 °C (8.5 °C in recent years) and the average annual precipitation is about 650 mm. The forest area lies at an altitude of 345–501 m. The area has been excluded from management activities since 1955. The area is used to conserve natural beech forests to support regional biodiversity, as well as for research purposes, e.g., to monitor spontaneous regeneration and the dynamics of stand structure^18, 49^.

### Design of study

The developmental stages of natural beech forest within the small developmental cycle were identified in permanent research plot in study area (Fig. 1). These permanent research plots, each with an area of 1 ha (100 × 100 m), were established in the period 1980 with aim to describe growth and development of old beech stands^49^. Based on these data, it is possible to classify parts of the stands into developmental stages. In this study, each developmental stage was represented by 2 plots on which 5 traps were regularly placed. Traps were at a minimum distance of 25 m from the nearest adjoining trap.

**Figure 2:**
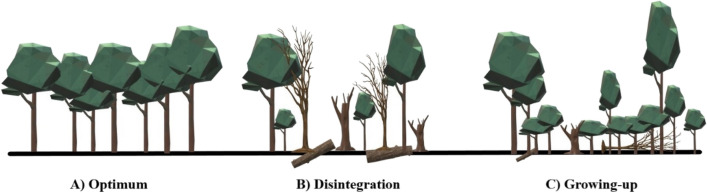
Schematic drawing of the developmental stages of beech forests with their typical structure. The schematic drawing was created in the Paint 3D application (Microsoft, Win10).

The development stages: (A) the optimum stage; (B) the disintegration stage; and (C) the growing-up stage on based typicall atributes according^46, 47^. Each stage is characterized by specific forest stand characteristics: (A) Optimum stage—high stock volume, low number of trees, horizontal canopy. (B) Disintegration stage—canopy break down, tree mortality, large volume of deadwood, reduction of stand stock. (C) Growing-up stage—large number of trees per area, majority of young trees, autoreduction of young stands, vertical canopy - the crowns touch each other and interpenetrate in the vertical direction (Fig. 2). The study area is situated inside a large forest complex, thus avoiding edge effects on beetle communities.

For this research, input environmental variables were quantified (Table 1) on the research plots in the close proximity of monitoring samples (radius 10 m, area 314 m^2^). This included all lying dead wood from diameter d > 7 cm volumes measured as l × g_1,2_ (length × basal area at half the length of the log) and standing dead wood from size d > 7 cm; h > 1.3 m volumes measured as π × d_1.3_^2^ (3.14 × diameter at breast height^2^). The dead woods dimensions obtained by manual measurement. Dead wood was then converted to m^3^/ha. Furthermore, the number of microhabitats was summarized according to the methodology^36^. Plant cover and natural renewal (generation) was assessed as a percentage of the buffer around the trap. Tree canopy openness was assessed by analyzing hemispherical photographs (fisheye lens) using Gap Light Analyzer 2.0. The program analyzes the percentage of light transmission through the tree canopy to the ground at the trap location and converts it into % values.

**Table 1:** Table showing the variables surveyed by stage.

		Optimum	Disintegration	Growing up
Deadwood	m^3^/ha	**13.7** (0–95) ± 30.39	**92.2** (0–207) ± 86.5	**40.1** (0–143) ± 53.5
Microhabitats	pc/plot	**1.3** (0–4) ± 1.25	**7.4** (1–20) ± 5.6	**5.2** (0–30) ± 8.89
Generation	%	**5.0** (0–30) ± 9.4	**48.5** (10–80) ± 23.1	**52.5** (0–95) ± 30.4
Plants	%	**2.5** (0–5) ± 2.6	**14.5** (5–45) ± 13.0	**3.5** (0–15) ± 5.3
Canopy openness	%	**5.9** (2.9–9.5) ± 1.84	**13.2** (6.9–22.5) ± 5.24	**9.5** (5.3–15.0) ± 2.9

The mean value is marked in bold. In brackets is range (minimun-maximum) and followed by the Std. Dev.

### Beetle sampling

**Figure 3:**
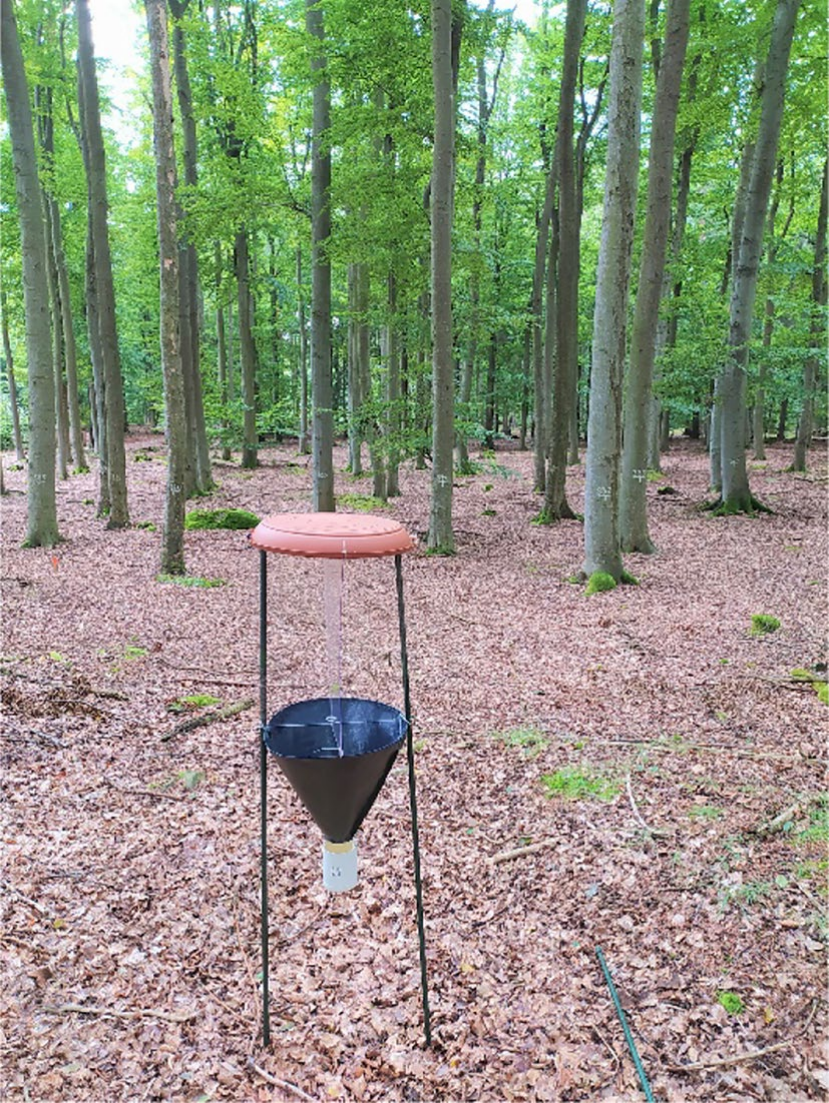
Passive interception flight traps located in optimum stage of beech forest.

The model group for the study was the order Coleoptera. Data were collected during April–September 2021 using 30 passive interception flight traps (unbaited). Traps were picked every 2–3 weeks. Each traps consisted of a roof, plexiglass barrier, funnel, and collection container. The roof of the trap was made of a plastic dish that was 45 cm in diameter. Underneath the roof, there were two plexiglass panes perpendicular to one another, forming a 40 × 50 cm (width × height) barrier (Fig. 3). The preservative was propylene glycol solution (1:1.5) with a drop of degreasing agent as detergent. Traps were placed on poles at 1.5 m above the ground (Fig. 3) representing the optimal flight height of the beetles^50^. Ten monitoring samples were placed at each developmental stage. The collected material were determined into species level, except for the family Staphylinidae due to problematic determination. However, the absence of this family will not affect the final assessment, because it is highly correlated with other beetle species^51^. Species were classified follow by list Schmidl and Bußler^52^, and Seibold et al.^53^ into saproxylic species and non-saproxylic. Saproxylic beetles are depend on deadwood of all types and sizes and also on other organisms living on deadwood, e.g., mycetophages on tree fungi or carnivor to other obligate saproxylic species. Species were ranked according to their degree of threat according to the Red-List of Endangered Species of the Czech Republic, Invertebrates^54^. The taxonomy of species corresponds to the concept of Zich O. (ed.) (2022) BioLib. http://www.biolib.cz.

## Data analysis

One-way analysis of variance ANOVA was used to compare differences in species richness of saproxylic, non-saproxylic and endangered species (mean observed richness per trap) between the three developmental stages of natural beech stands. Verification of normality was performed by Shapiro–Wilk tests (no normality distribution for endangered species group—was used nonparametric ANOVA Kruskal–Wallis test). The post hoc Tukey HSD method was used to compare differences between stages. For all group was checked the homogenity of variance using Bartlett test According by^55^. Analysis were perfomed in Statistica 13 software (StatSoft, Inc.)

In addition, species accumulation curves were generated by classic formula for Chao1^56, 57^ was used to estimate the total site number of beetle species. Analyses were computed in the EstimateSWin910 software with the number of randomizations 1000^58^. Rarefaction- extrapolation approach that estimated the rate of increase in the number of species per number of samples, was used similarly to Seibold et al.^59^. Curves were generated to compare developmental stages with each other based on Chao et al. Chao et al.^60^ in the Inext program Chao et al.^61^. Data were calculated based on incidence data with 200 bootstraps, and 3 sets of cumulative curves were calculated according to Hill’s numbers q = 0 (species richness), q = 1 (exponential of Shannon’s entropy index), and q = 2 (inverse of Simpson’s concentration index). Hill numbers offer some distinct advantages over other diversity indices^60^. The method of non-overlapping confidence intervals was used for the significance of diversity curves^62^.

Redundancy analysis (RDA) with log transformation using the all constrained canonical axes test was used to monitor the significance of species preferences by stage distribution. This analysis used a scheme of Van Dobben circles (T value biplots) to test the significance of a specific species preference on the explained variable. All ordination calculations were with 4499 unrestricted Monte Carlo permutations. Canonical corespondence analysis (CCA) was used for dimensional representation of species richness on a categorical variable development stage, was used split-plot design (restricted 4499 Monte Carlo permutations) with the whole plot freely exchangeable, according^63^. RDA(log) method to test individual canonical axes, it was determined which environmental variables explained the greatest variability from the observed sample of captured species separately by stage—summarized the effects of explanatory variables using a Monte Carlo permutation test of significance of variables separately for each group of beetles. All ordination calculations were with 4499 unrestricted Monte Carlo permutations All ordination calculations were performed in the CANOCO 5 program for multivariate statistical analysis using ordination methods^64, 65^.

## Results

### Study site

**Figure 4:**
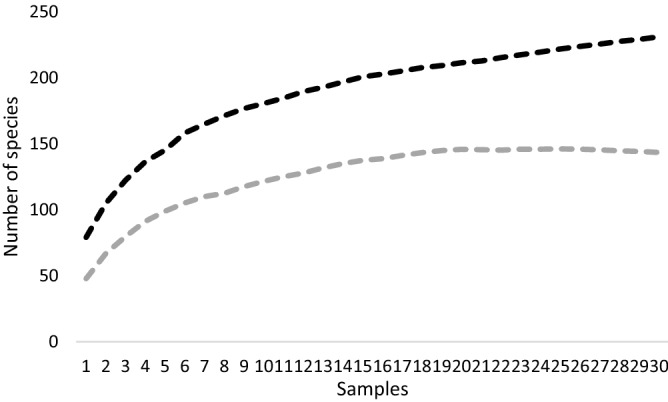
Total species estimates of study site by Chao1. The curves show the accumulation of saproxylic (black) and non-saproxylic (grey) species at the site.

A total of 290 species were recorded (supplementary 4S), with a total number of 8380 individuals. The species of 26 adults could not be identified. Saproxylic species totaled 177 (61%), and 113 species (39%) were non-saproxylic. There were 22 saproxylic species from the Red-List, and no non-saproxylic endangered species were found. Estimates of the maximum numbers of species (Chao1) inhabiting the study site varied considerably by beetle group, with an increase of probably 31% (54 + species) estimated for saproxylic species and an increase of 28% (32 + species) for non-saproxylic species. For this group (non-saproxylic), this sum of species is probably the maximum for the locality (Fig. 4).

### Developmental stages

**Figure 5:**
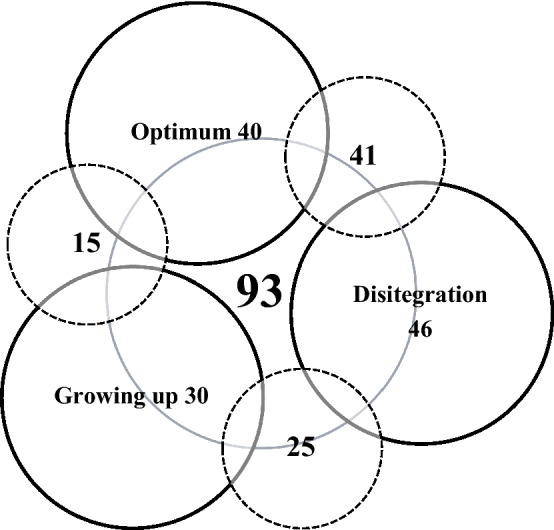
Venn diagram – shows the absolute recorded unique numbers of species in each stage (solid circles). Between each stage are the species that are shared within the stages (dashed circles) and in the middle is the sum of species shared for all stages simultaneously.

Absolute numbers of species recorded in development stages are shown in the Venn diagram (Fig. 5).

**Figure 6:**
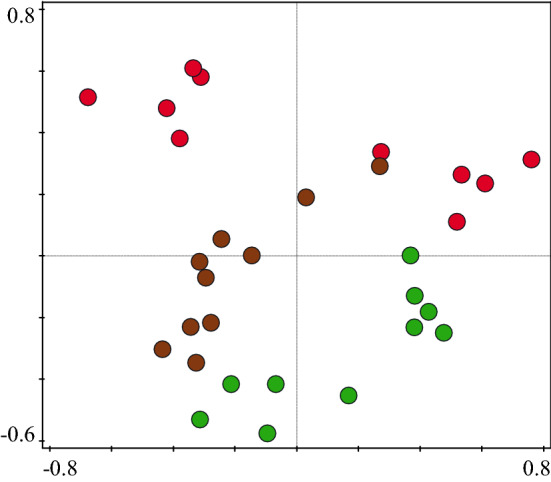
PCoA summarize the beetle species communities similarity/dissimilarity by development stage of beech stands. Red circle indicates optimum stage. Brown cicrle indicates disintegration stage and Green circle indicates growing up stage.

**Figure 7:**
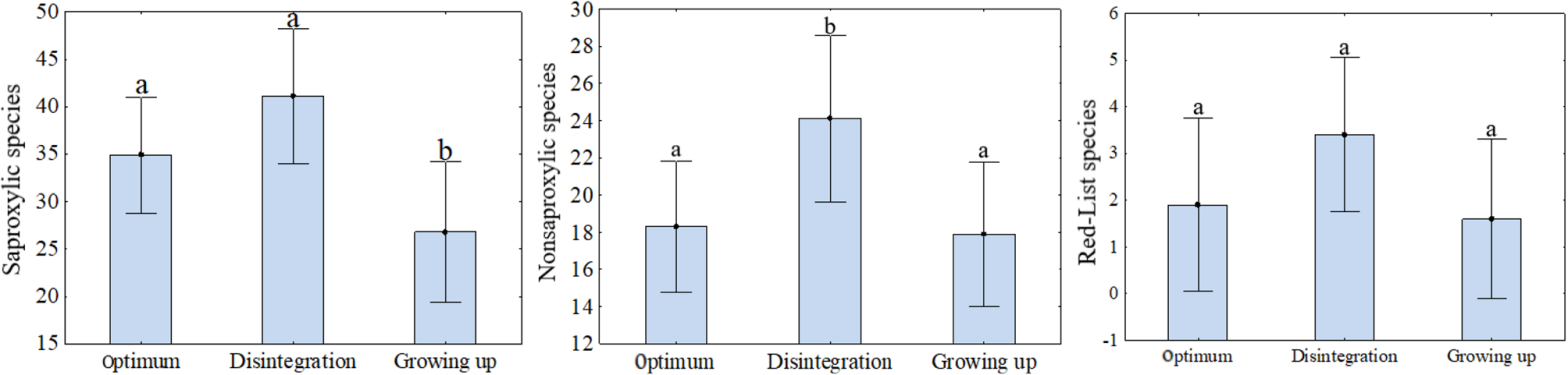
Mean number of beetle species by stage over the study period. According to beetle group (1) saproxylic species (2) non-saproxylic species (3) red-listed species. Vertical error bars denote standard deviation (SD), end of chart mean (mean ± SD) Letters above error bars indicated differences by Tukey HSD Post Hoc test.

PCoA diagram showed different composition of beetle communities within development stages (Fig. 6). Within the group of saproxylic F(2;27) = 10.7514; *p* = 0.0004) and non-saproxylic species F(2;27) = 7.5971; *p* = 0.0024, a significant difference between the stages was detected. For the endangered species, the difference between stages was on the edge of significance KW-H(2;30) = 5.4687; *p* = 0.0649) (Fig. 7). Multiple comparisons showed that the disintegration phase was tendency richer for all dependent variables (Fig. 7).

**Figure 8:**
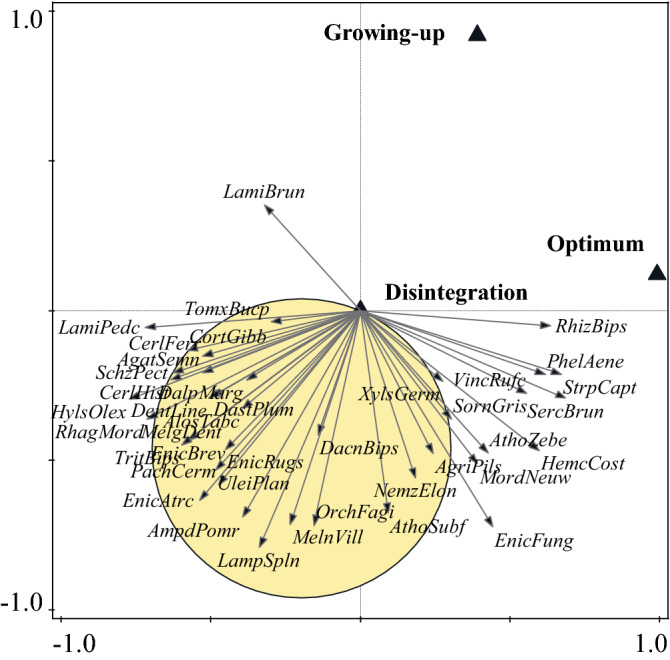
Van Dobben (T value biplot): visualization of overall species preference at stages of autonomous development of beech stands. Test of all canonical axes (pF 2.8; *p* = 0.002). The test was performed from the entire list of recorded species (N > 1), and the 40 best-fit species were selected for visualization. Species whose arrow ends in a yellow circle are significantly associated with the disintegration stage.

**Figure 9:**
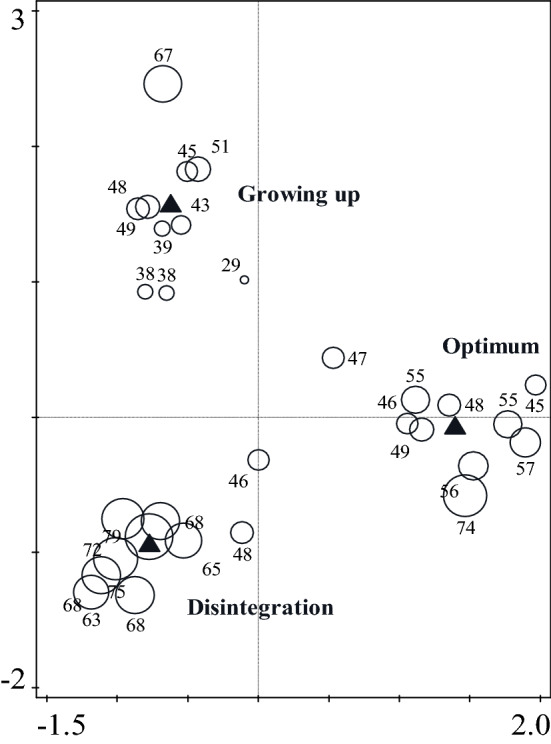
Diagram of sample by development stage of beech stands, which the size of each circle indicates the species richness of the samples (absolute number of species in the trap) CCA (pF = 1.3; *p* =  < 0.001).

**Figure 10:**
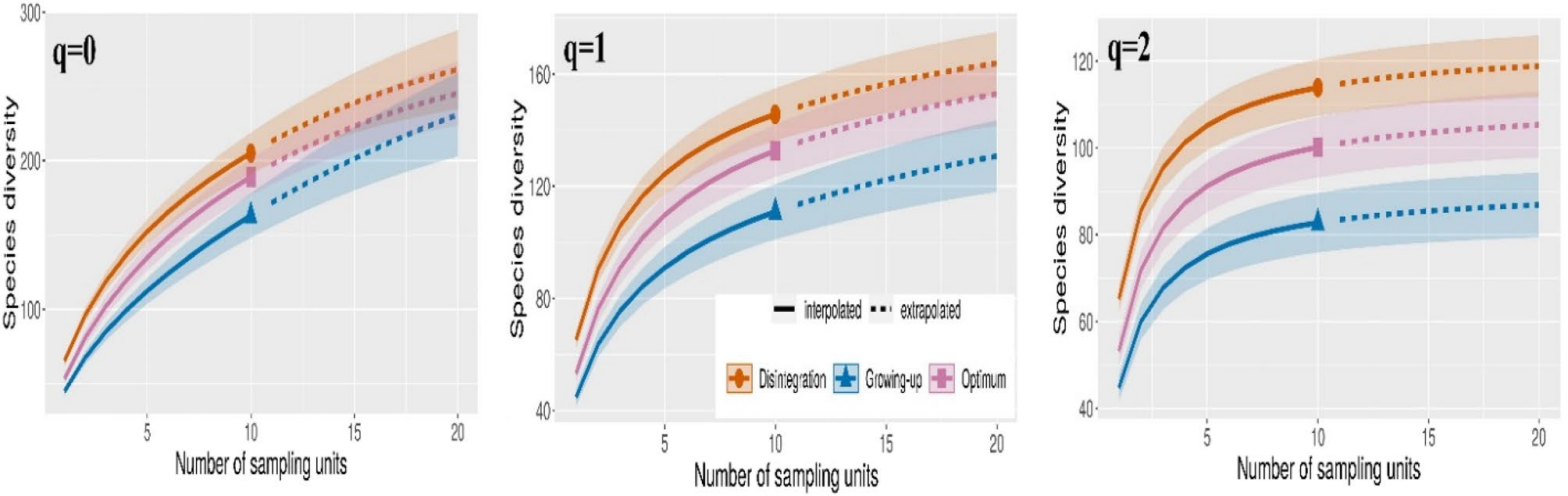
Species gamma diversity curves split separately by cycles divided into Hill’s numbers q = 0 (species richness), q = 1 (exponential of Shannon’s entropy index), and q = 2 (inverse of Simpson’s concentration index). The curves were extrapolated by the same number of samples, the colored shaded areas are the 95% confidence interval. Solid symbols represent the total number of study samples.

The species preference for developmental stages according to RDA is exclusively directed to the disintegration stage and partially to the optimum stage (Fig. 8). Similarly, traps located in disintegration stage captured a much higher number of species compared to other developmental stages (Fig. 9). Diversity indices according to Hill’s numbers (q = 0,1,2) were also highest in the disintegration stage, non-overlapping confidence intervals especially compared to growing up phase(Fig. 10). Saproxylic species dominated the stages 59–67% and thus did not show large proportional differences. The lowest ratio of saproxylic to non-saproxylic species occurred in the disintegration stage and the highest in the optimum stage. The occurrence of Red-Listed species ranged from 6 to 8% of the total number of species.

At each stage, a different environment was created. Redundancy analysis (RDA) was used to find the variables that best explained the species preference. At the optimum stage, a single significant variable was found—plant cover for both groups of beetles (Table 2, Fig. 1S). This was in contrast to the disintegration stage, where the number of microhabitats and the volume of deadwood were conclusively positive. For non-saproxylic species, plant cover was borderline significant (Table 2, Fig. 2S). As the development progressed, the canopy openness, deadwood, and microhabitats, which preferred saproxylic species, became significantly more pronounced at the growing-up stage. For non-saproxylic species, canopy openness explained most of the preference (Table 2, Fig. 3S).

**Table 2:** Table of variables that best explained species preference in each stage separately.

	Saproxylic species	Non-saproxylic species
Optimum	Plants (28.3%; pF 3.2; *p* = 0.01)	Plants (50.6%; pF 8.2; *p* = 0.01)
Disintegration	Microhabitats (16.2%; pF 1.5; *p* = 0.01)	Plants (15.5%; pF 1.5; *p* = 0.07)
	Deadwood (15.2%; pF 1.4; *p* = 0.05)	
Growing up	Canopy openness (22.0%; pF 2.3; *p* = 0.05)	Canopy openness (24.1%; pF 2.5; *p* = 0.05)
	Microhabitats (19.3%; pF 1.9; *p* = 0.05)	
	Deadwood (18.8%; pF 1.9; *p* = 0.05)	

## Discussion

### Study site

In the recently unmanaged beech forests, 177 saproxylic species (Chao1 estimate up to 231 species) were observed, slightly over 60% of the total number of species. This is consistent with the percentage values found by Parisi et al.^66^ and supports the assumption that saproxylic beetle species make up the majority of beetle biodiversity in forests. This relatively high number of species demonstrates that beech forests are of high conservation value for this group of beetles. In European beech forests, the number of saproxylic species is estimated at 800–860^15^. In comparison with Müller et al.^15^, between 20 and 22% of the total saproxylic beetle species richness was observed in this study (Chao1 estimates up to 27–29%). Flight interception traps are an effective tools for evaluating beetle biodiversity in the landscape. This type of trap captures local beetle species effectively^67, 68^ and is an appropriate method for comparisons between different forest habitats^69^. Flight intercept traps are a highly complementary method of beetle monitoring to wood incubation comparisons, and thus well capture the true density of beetle communities, not only capturing the more active species^70, 71^.

In general, beetles have a limited dispersal potential due to their long larval development and the short-term stage of actively dispersing adults. Species dispersal is time-limited, and having suitable deadwood to colonize during this period is essential^72^. Indeed, in terms of life-history strategies, saproxylic species are not very dispersive^66, 73^. New suitable sites are colonized very slowly by endangered species because they have limited mobility^74^. Particularly important is the group of so-called forest relicts, which needs uninterrupted habitat continuity and large amounts of deadwood^75^. For these reasons, saproxylic species are considered bioindicators of forest ecosystems^76^ and are therefore the most studied insect group^11^ and one of the priority groups in conservation strategies^12^.

### Developmental stages

We detected natural dynamics as an important driver of species richness in forests. Beetles are sensitive to environmental change, but this may also be due to management activities, especially in terms of insolation^77^. On the other hand, they respond negatively to the removal of all deadwood, e.g., Thorn et al.^7^, Zumr and Remeš^78^. Each developmental stage creates different natural conditions, and therefore, beetles respond differently to each stage^79^. The disintegration stage is the early successional stage of the newly forming beech stand. In this study, areas of early disturbance were confirmed to be sites of high beetle richness^4, 39, 80^. However, this phase lasts for a relatively short time, typically up to 20 years^47^, after which species richness declines rapidly, according to our observations. Compared to large-scale disturbance, however, this period of high diversity seems to last longer in small-scale disturbances. Declines in invertebrate species and reductions in plant and tree richness have been observed as early as 3–5 years after the onset of large-scale disturbances^45, 79^. Small-scale dynamics may be more favorable to beetles, as the patch is generally not disturbed enough to homogenize the entire area with a strong successional cover of herbs and shrubs, supporting the thesis^81^. In the disintegration stage, the higher species diversity was mainly due to the presence of deadwood and microhabitats in our analysis. The importance of microhabitats was thus confirmed^19, 36^. In protected beech stands, the most common wood-boring fungus is *Fomes fomentarius*^82^. The most abundant microhabitats in our study were wood-decay fungi, especially of the genus *Fomes*, which are very important microhabitats for saproxylic beetles^83^.

The role of deadwood for saproxylic beetle species richness has also been confirmed^84, 85^. The natural small-scale dynamics of development creates structurally heterogeneous stands, which is important for beetle diversity^75, 86^. Due to its complexity, small-scale disturbances appear to be preferable and able to substitute large-scale disturbances^81^. At different stages, plant cover, among others, was found to be the best explanatory variable for both saproxylic and non-saproxylic species. For saproxylic species, this could be an extension of the food supply of floricuolus species. Non-saproxylic species are much more plant-associated, as they are generally plant or plant root eaters. For example, the family Nitidulidae and its genera *Brassicogethes*^87^ and *Lamiogethes*^87^ are dependent on forest plants of the *Brassicaceae* B. and *Lamiaceae* L. The larvae of *Strophosoma melannogramum* (Forster, 1771) of the family Curculinoidae feed on roots of the genera *Rumex* L. and *Deschampsia* L.^88^. Also, species of the genus *Athous* (Esch. 1829) devour roots, and some species have trophism similar to the genus *Dalopius* (Esch. 1829), whose predatory larvae seek food in the root system of herbs. The genus *Agriotes* (Esch. 1829) feeds directly on the roots of herbs^89^. The investigated significance of plant cover in our study can be confirmed by the importance for a wider range of non-saproxylic species, as also found for the non-saproxylic Carabidae and Staphylinidae by the authors^90^. Beech stands enter a stage of disintegration at around 250 years of age^47^. The spatiotemporal formation of new suitable conditions for beetles in the growing-up stage can be complicated, as beech wood decomposes rapidly, and even large logs decompose within 50 years^91, 92^. Under dense natural regeneration, highly decomposed deadwood can occur as early as the initial period of the growing-up stage. The invertebrates inhabiting this substrate tend to be shade-tolerant species^93^, usually earthworms (Lumbricidae), and the beetles are mainly the epigeic, usually non-saproxylic Carabidae and Staphylinidae^94^. In beetles, it is observed that some species have adapted to tree shade and are more shade tolerant^95, 96^. For example, oak (*Quercus* sp.) grows in the warm lowland parts of Europe. Saproxylic beetle species living on oak, therefore, respond sensitively and positively to the increase in temperature^97^ and are therefore strongly influenced by the canopy openness^98, 99^. The importance of insolation for beetle diversity is far more significant in oak forests than in beech forests, especially for the threatened species^100^. Nevertheless, it was confirmed in this study that insolation is an important factor in increasing beetle diversity also in beech stands^33, 71, 101, 102^. Other invertebrates, such as spiders^103^ and Carabidae^101, 104, 105^. In the disintegration stage, there were not only more beetle species but also more adult individuals recorded in this study. Compared to the optimum stage, the beetle abundance was twice as high, and when compared to the growing-up stage, the abundance was almost 1.5 times higher. This finding is consistent with the assumption that invertebrate biomass increases linearly with higher temperatures (caused by canopy openness). However, the positive effect of temperature increase for invertebrate biomass growth has its limits and leads to a decrease in biodiversity in cases of temperature increase beyond the optimum^106^. From the perspective of climate change adaptation, a small-scale silvicultural scheme seems to be the best option^107^. This, when supporting beetle feeding substrates in managed forests using small-scale restoration elements, can lead to the creation of adapted resilient forest stands with widely advanced biodiversity.

## Conclusion

This research confirmed that the reserve, although recently established, may be a suitable habitat for saproxylic beetles. Some species have been found and confirmed from sites many tens of kilometers away. Thus, it can be inferred that the study area has supported several microhabitats in the past where these rare beetles have survived, and after the establishment of the reserve, many suitable microhabitats have occurred, and where the species have naturally proliferated and are easier to detect. The results obtained from this research allow us to answer the research questions that were defined in the introduction.

1. It has been confirmed that the natural small-scale dynamics of native stand development creates many essential habitats for saproxylic beetles, and the highest species diversity has been documented in the disintegration stage. Thus, we do not give weight to the time period since the establishment of the reserve according to Paillet et al.^12^, but we are inclined toward the argument^19, 34^ that what is more important is how quickly suitable conditions for beetle life are established in the stands.
2. The synergy of food and habitat heterogeneity enhanced canopy openness and has been shown to create the conditions for an increase in saproxylic beetle biodiversity^45, 73^. Plant cover was another important variable for non-saproxylic species. If the goal is to increase species diversity on a broader landscape scale, management of commercial, managed forests must be modified in addition to the establishment of reserves (the extent of which faces economic and social limits). As a non-management conservation strategy, it can be very inappropriate for many beetle species, especially sun-preferring species^53^. Any preference for one management method leads to a substantial homogenization of regional biodiversity^108^. Solutions can be sought in integrative management^23, 109^, creating suitable ecological niches through deadwood enrichment. There, rarer species from small-area reserves that abound with high volumes of deadwood may gradually spread^110^. It is also advisable to maintain a mosaic of habitats with specific forms of management, e.g., open sunny pasture forests, which are extremely unique in biodiversity^111^ as well as, e.g., coppice forests^112^.

## Supplementary Information

Supplementary Information.

## Data Availability

All data generated or analysed during this study are included in this published article [and its supplementary information files.
