# Supplementary material for: Small-scale spontaneous dynamics in temperate beech stands as an importance driver for beetle species richness
Source: Sci Rep. 2022 Jul 13;12:11974. doi: 10.1038/s41598-022-16352-7 (PMC9279280; doi:10.1038/s41598-022-16352-7)
Supplement: Supplementary file 1 — Supplementary Information. [file 41598_2022_16352_MOESM1_ESM.docx]

Fig. 1S. Optimum stage. Left, saproxylic-species diagram: the first constrained axis explained 29.65% (pF 1.7, p = 0.058), the second, 11.26% (pF 0.8, p > 0.05). Right, non-saproxylic species diagram: the first constrained axis explained 50.72% (pF 4.1, p < 0.01), the second, 9.68% (pF 1.0, p > 0.05). Tests were performed from the entire list of recorded species (N>1), with the 40 best fitting species for visualization.

Fig. 2S. Disintegration stage. Left, saproxylic-species diagram: the first constrained axis explained 16.74% (pF 0.8, p > 0.05), the second, 16.02% (pF 1.0, p > 0.05). Right, non-saproxylic species diagram: the first constrained axis explained 21.22% (pF 1.1, p > 0.05), the second, 14.12% (pF 0.9, p > 0.05). Tests were performed from the entire list of recorded species (N > 1), with the 40 best fitting species for visualization.

Fig. 3S. Growing-up stage. Left, saproxylic species diagram: the first constrained axis explained 31.8% (pF 1.9, p < 0.05), the second, 13.44% (pF 1.0, p > 0.05). Right, non-saproxylic species diagram: the first constrained axis explained 34.92% (pF 2.1, p < 0.01), the second, 11.73% (pF 0.9, p > 0.05). Tests were performed from the entire list of recorded species (N > 1), with the 40 best fitting species for visualization.

4S) List of species collected in developmental stages in beech stands. Saproxylic (SPX) and non-saproxylic (N-SPX) species were classified according to (Schmidl & Bußler, 2004; Seibold et al., 2015b). The threat status: critically endangered (CR), endangered (EN) vulnerable (VU) and near threatened (NT) was classified according to (Hejda et al., 2017)

|  |  |  | **Threat status** | **Growing up** | **Optimum** | **Disintegration** |
| --- | --- | --- | --- | --- | --- | --- |
| **Family** | **Species** |  |  |  |  |  |
| **Aderidae** | *Euglenes oculatus* (Paykull, 1798) | SPX |  | 1 |  | 1 |
| **Anthribidae** | *Anthribus nebulosus* (J.R. Forster, 1771) | N-SPX |  |  | 2 | 1 |
|  | *Dissoleucas niveirostris* (Fabricius, 1798) | SPX |  |  | 1 |  |
|  | *Platystomos albinus* (Linnaeus, 1758) | SPX |  |  | 1 | 3 |
| **Attelabidae** | *Lasiorhynchites olivaceus* (Gyllenhal, 1833) | N-SPX |  |  | 1 |  |
| **Buprestidae** | *Agrilus viridis* (Linnaeus, 1758) | SPX |  | 1 |  |  |
| **Byturidae** | *Byturus ochraceus* (L. G. Scriba, 1790) | N-SPX |  | 1 | 2 | 2 |
| **Cantharidae** | *Cantharis nigricans* (O.F. Müller, 1776) | N-SPX |  |  | 1 |  |
|  | *Cantharis obscura* (Linnaeus, 1758) | N-SPX |  | 2 | 3 | 4 |
|  | *Malthinus flaveolus* (Herbst, 1786) | SPX |  | 1 |  |  |
|  | *Rhagonycha lignosa* (O.F. Müller, 1764) | N-SPX |  | 8 | 14 | 13 |
| **Carabidae** | *Acupalpus flavicollis* (Sturm, 1825) | N-SPX |  |  | 1 | 1 |
|  | *Amara ovata* (Fabricius, 1792) | N-SPX |  | 1 | 1 | 3 |
|  | *Anchomenus dorsalis* (Pontoppidan, 1763) | N-SPX |  | 1 | 1 |  |
|  | *Badister bullatus* (Schrank, 1798) | N-SPX |  | 1 |  |  |
|  | *Dromius quadrimaculatus* (Linnaeus, 1758) | N-SPX |  |  |  | 1 |
|  | *Harpalus distinguendus* (Duftschmid, 1812) | N-SPX |  | 1 |  |  |
|  | *Notiophilus biguttatus* (Fabricius, 1779) | N-SPX |  |  | 1 |  |
|  | *Notiophilus rufipes* (Curtis, 1829) | N-SPX |  |  |  | 1 |
|  | *Ocydromus deletus* (Audinet-Serville, 1821) | N-SPX |  | 1 |  |  |
|  | *Ophonus melletii* (Heer, 1837) | N-SPX |  |  |  | 1 |
|  | *Pseudoophonus rufipes* (De Geer, 1774) | N-SPX |  |  |  | 1 |
|  | *Trechus quadristriatus* (Schrank, 1781) | N-SPX |  | 4 | 2 | 1 |
| **Cerambycidae** | *Alosterna tabacicolor* (DeGeer, 1775) | SPX |  | 2 | 3 | 21 |
|  | *Anaglyptus mysticus* (Linnaeus, 1758) | SPX |  |  | 4 | 3 |
|  | *Anoplodera sexguttata* (Fabricius, 1775) | SPX |  |  | 3 | 5 |
|  | *Clytus arietis* (Linnaeus, 1758) | SPX |  |  | 1 | 3 |
|  | *Leptura quadrifasciata* (Linnaeus, 1758) | SPX |  | 1 | 4 | 2 |
|  | *Obrium brunneum* (Fabricius, 1793*)* | SPX |  | 2 | 4 | 2 |
|  | *Oxymirus cursor* (Linnaeus, 1758) | SPX |  |  | 1 |  |
|  | *Judolia cerambyciformis* (Schrank, 1781) | SPX |  |  | 1 | 4 |
|  | *Paracorymbia maculicornis* (DeGeer, 1775) | SPX |  |  |  | 2 |
|  | *Prionus coriarius* (Linnaeus, 1758) | SPX |  | 4 | 6 | 3 |
|  | *Rhagium mordax* (DeGeer, 1775) | SPX |  | 6 | 6 | 13 |
|  | *Stenurella melanura* (Linnaeus, 1758) | SPX |  | 8 | 19 | 11 |
|  | *Stictoleptura rubra* (Linnaeus, 1758) | SPX |  | 1 | 2 |  |
|  | *Leptura maculata* (Poda, 1761) | SPX |  | 1 |  | 1 |
|  | *Tetropium castaneum* (Linnaeus, 1758) | SPX |  |  | 1 |  |
| **Cerylonidae** | *Cerylon fagi* (Brisout de Barneville, 1867) | SPX |  | 9 | 3 | 9 |
|  | *Cerylon ferrugineum* (Stephens, 1830) | SPX |  | 20 | 11 | 47 |
|  | *Cerylon histeroides* (Fabricius, 1792) | SPX |  | 14 | 7 | 32 |
| **Ciidae** | *Cis bidentatus* (Olivier, 1790) | SPX |  |  |  | 2 |
|  | *Cis castaneus* (Herbst, 1793) | SPX |  |  | 1 |  |
|  | *Cis fagi* (Waltl, 1839) | SPX |  | 2 | 7 | 7 |
|  | *Cis festivus* (Panzer, 1793) | SPX |  |  |  | 1 |
|  | *Cis glabratus* (Mellié, 1848*)* | SPX |  |  |  | 1 |
|  | *Cis micans* (Fabricius, 1792) | SPX |  | 16 | 27 | 22 |
|  | *Cis rugulosus* (Mellié, 1848) | SPX |  |  | 2 |  |
|  | *Octotemnus glabriculus* (Gyllenhal, 1827) | SPX |  |  |  | 1 |
|  | *Orthocis alni* (Gyllenhal, 1813) | SPX |  |  | 1 |  |
|  | *Orthocis lucasi* (Abeille de Perrin, 1874) | SPX |  |  | 1 | 2 |
|  | *Sulcacis fronticornis* (Panzer, 1809) | SPX |  | 1 | 1 | 3 |
|  | *Sulcacis nitidus* (Fabricius, 1792) | SPX |  |  | 5 | 4 |
| **Cleridae** | *Thanasimus formicarius* (Linnaeus, 1758) | SPX |  |  | 1 |  |
|  | *Tillus elongatus* (Linnaeus, 1758) | SPX |  |  | 1 |  |
| **Coccinellidae** | *Adalia decempunctata* (Linnaeus, 1758) | N-SPX |  |  | 1 |  |
|  | *Coccinella septempunctata* (Linnaeus, 1758) | N-SPX |  |  |  | 1 |
|  | *Exochomus quadripustulatus* (Linnaeus, 1758) | N-SPX |  | 1 | 2 |  |
|  | *Nephus bipunctatus* (Kugelann, 1794) | N-SPX |  |  | 1 |  |
| **Cryptophagidae** | *Antherophagus nigricornis* (Linnaeus, 1758) | N-SPX |  |  |  | 1 |
|  | *Atomaria analis* (Erichson, 1846) | N-SPX |  |  |  | 1 |
|  | *Atomaria bella* (Reitter, 1875) | SPX |  |  | 1 |  |
|  | *Atomaria fuscata* (Schönherr, 1808) | N-SPX |  |  |  | 1 |
|  | *Atomaria linearis* (Stephens, 1830) | N-SPX |  |  |  | 2 |
|  | *Atomaria testacea* (Stephens, 1830) | N-SPX |  |  | 1 |  |
|  | *Atomaria turgida* (Erichson, 1846) | SPX |  |  | 1 |  |
|  | *Cryptophagus dentatus* (Herbst, 1793) | N-SPX |  |  | 3 | 4 |
|  | *Cryptophagus dorsalis* (C.R. Sahlberg, 1834) | SPX |  |  | 1 | 1 |
|  | *Cryptophagus labilis* (Erichson, 1846) | SPX |  | 1 | 1 | 1 |
|  | *Cryptophagus micaceus* (Rey, 1889) | SPX |  | 6 | 2 | 9 |
|  | *Cryptophagus pubescens* (Sturm, 1845) | N-SPX |  | 6 | 2 | 3 |
|  | *Cryptophagus punctipennis* (B. de Barneville, 1863) | N-SPX |  | 1 | 1 | 2 |
|  | *Cryptophagus scutellatus* (Newman, 1834) | N-SPX |  |  |  | 2 |
|  | *Micrambe abietis* (Paykull, 1798) | SPX |  | 1 | 2 |  |
| **Curculionidae** | *Acalles camelus* (Fabricius, 1792) | SPX |  |  | 1 |  |
|  | *Ceutorhynchus pallidactylus* (Marsham, 1802) | N-SPX |  |  | 3 | 1 |
|  | *Crypturgus cinereus* (Herbst, 1793) | SPX |  | 1 |  |  |
|  | *Curculio glandium* (Marsham, 1802) | N-SPX |  | 1 |  | 1 |
|  | *Dryocoetes autographus* (Ratzeburg, 1837) | SPX |  | 1 | 3 | 2 |
|  | *Ernoporicus fagi* (Fabricius, 1798) | SPX |  | 7 | 6 | 10 |
|  | *Hylastes cunicularius* (Erichson, 1836) | SPX |  | 2 | 4 |  |
|  | *Hylastes opacus* (Erichson, 1836) | SPX |  | 1 |  |  |
|  | *Ips typographus* (Linnaeus, 1758) | SPX |  | 1 |  | 1 |
|  | *Orchestes fagi* (Linnaeus, 1758) | N-SPX |  |  | 5 | 6 |
|  | *Phyllobius argentatus* (Linnaeus, 1758) | N-SPX |  | 4 | 5 | 3 |
|  | *Phyllobius maculicornis* (Germar, 1824) | N-SPX |  |  |  | 1 |
|  | *Pityogenes bidentatus* (Herbst, 1784) | SPX |  |  | 1 |  |
|  | *Pityogenes chalcographus* (Linnaeus, 1761) | SPX |  |  | 5 | 1 |
|  | *Pityophthorus pityographus* (Ratzeburg, 1837) | SPX |  | 1 | 3 | 1 |
|  | *Polydrusus tereticollis* (DeGeer, 1775) | N-SPX |  | 10 | 2 | 4 |
|  | *Polygraphus poligraphus* (Linnaeus, 1758) | SPX |  | 1 |  |  |
|  | *Ruteria hypocrita* (Boheman, 1837) | SPX |  |  | 5 | 1 |
|  | *Scolytus carpini* (Ratzeburg, 1837) | SPX |  |  | 1 | 1 |
|  | *Sitona lineatus* (Linnaeus, 1758) | N-SPX |  |  | 1 | 1 |
|  | *Sitona macularius* (Marsham, 1802) | N-SPX |  | 1 |  | 1 |
|  | *Stenocarus ruficornis* (Stephens, 1831) | N-SPX |  | 1 |  |  |
|  | *Stereocorynes truncorum* (Germar, 1824) | SPX |  | 1 | 1 | 1 |
|  | *Strophosoma capitatum* (De Geer, 1775) | N-SPX |  | 3 | 22 | 1 |
|  | *Strophosoma melanogrammum* (Forster, 1771) | N-SPX |  | 55 | 60 | 39 |
|  | *Taphrorychus bicolor* (Herbst, 1793) | SPX |  | 68 | 54 | 69 |
|  | *Trypodendron domesticum* (Linnaeus, 1758) | SPX |  | 3 | 2 | 1 |
|  | *Tychius picirostris* (Fabricius, 1787) | N-SPX |  | 1 |  | 1 |
|  | *Anisandrus dispar* (Fabricius, 1792) | SPX |  |  | 2 |  |
|  | *Xyleborus monographus* (Fabricius, 1792) | SPX |  |  |  | 2 |
|  | *Xylosandrus germanus* (Blandford, 1894) | SPX |  | 33 | 134 | 62 |
| **Dermestidae** | *Anthrenus museorum* (Linnaeus, 1761) | N-SPX |  |  |  | 2 |
|  | *Dermestes murinus* (Linnaeus, 1758) | N-SPX |  | 1 |  |  |
|  | *Globicornis nigripes* (Fabricius, 1792) | SPX |  |  |  | 1 |
|  | *Megatoma undata* (Linnaeus, 1758) | SPX |  |  | 3 | 2 |
| **Elateridae** | *Agriotes pilosellus* (Schönherr, 1817) | N-SPX |  | 1 | 11 | 7 |
|  | *Agriotes sputator* (Linnaeus, 1758) | N-SPX |  |  |  | 1 |
|  | *Agrypnus murinus* (Linnaeus, 1758) | N-SPX |  | 1 | 1 | 2 |
|  | *Ampedus balteatus* (Linnaeus, 1758) | SPX |  |  | 4 |  |
|  | *Ampedus erythrogonus* (P.W.J. Müller, 1821) | SPX |  | 1 |  |  |
|  | *Ampedus nigrinus* (Herbst, 1784) | SPX |  |  | 2 | 1 |
|  | *Ampedus pomorum* (Herbst, 1784) | SPX |  | 1 | 3 | 6 |
|  | *Anostirus castaneus* (Linnaeus, 1758) | SPX |  |  |  | 2 |
|  | *Anostirus purpureus* (Poda, 1761) | SPX |  | 1 |  | 1 |
|  | *Athous haemorrhoidalis* (Fabricius, 1801) | N-SPX |  | 3 | 2 | 5 |
|  | *Athous subfuscus* (O.F. Müller, 1767) | N-SPX |  | 128 | 375 | 301 |
|  | *Athous vittatus* (Fabricius, 1792) | N-SPX |  | 30 | 18 | 44 |
|  | *Athous zebei* (Bach, 1854) | N-SPX |  | 21 | 192 | 44 |
|  | *Cardiophorus nigerrimus* (Erichson, 1840) | N-SPX |  | 1 |  | 1 |
|  | *Dalopius marginatus* (Linnaeus, 1758) | N-SPX |  | 70 | 77 | 175 |
|  | *Denticollis linearis* (Linnaeus, 1758) | SPX |  | 2 | 1 | 11 |
|  | *Hemicrepidius niger* (Linnaeus, 1758) | N-SPX |  |  | 1 |  |
|  | *Melanotus castanipes* (Paykull, 1800) | SPX |  | 4 | 9 | 14 |
|  | *Melanotus villosus* (Geoffroy in Fourcroy, 1785) | SPX |  | 12 | 23 | 28 |
|  | *Nothodes parvulus* (Panzer, 1799) | N-SPX |  | 1 | 1 | 3 |
|  | *Omalisus fontisbellaquaei* (Geoffroy, 1785) | N-SPX |  |  | 1 | 1 |
|  | *Pheletes aeneoniger* (DeGeer, 1774) | N-SPX |  | 3 | 38 | 2 |
|  | *Procraerus tibialis* (Lac.Bois & Lacord, 1835*)* | SPX |  | 1 |  |  |
|  | *Selatosomus aeneus* (Linnaeus, 1758) | N-SPX |  |  |  | 1 |
| **Endomychidae** | *Endomychus coccineus* (Linnaeus, 1758) | SPX | VU | 1 |  |  |
|  | *Mycetina cruciata* (Schaller, 1783) | SPX |  | 2 | 1 |  |
| **Erotylidae** | *Dacne bipustulata* (Thunberg, 1781) | SPX |  | 13 | 53 | 112 |
|  | *Triplax aenea* (Schaller, 1783) | SPX |  |  | 12 | 2 |
|  | *Triplax lepida* (Faldermann, 1837) | SPX |  | 4 | 6 | 1 |
|  | *Triplax rufipes* (Fabricius, 1787) | SPX |  |  | 4 | 4 |
|  | *Tritoma bipustulata* (Fabricius, 1775) | SPX |  |  |  | 4 |
| **Eucnemidae** | *Clypeorhagus clypeatus* (Hampe, 1850) | SPX |  | 1 |  |  |
|  | *Hylis cariniceps* (Reitter, 1902) | SPX | CR | 1 | 1 | 1 |
|  | *Hylis foveicollis* (C.G. Thomson, 1874) | SPX | EN |  |  | 1 |
|  | *Hylis olexai* (Palm, 1955) | SPX | EN | 2 |  | 7 |
|  | *Isorhipis melasoides* (Laporte de Castelnau, 1835) | SPX | EN |  |  | 1 |
|  | *Melasis buprestoides* (Linnaeus, 1761) | SPX |  | 4 | 1 | 6 |
|  | *Microrhagus lepidus* (Rosenhauer, 1847) | SPX | EN |  | 1 |  |
| **Geotrupidae** | *Anoplotrupes stercorosus* (Hartmann, 1791) | N-SPX |  | 1 |  | 1 |
| **Histeridae** | *Abraeus granulum* (Erichson, 1839) | SPX | VU | 1 | 1 |  |
|  | *Paromalus flavicornis* (Herbst, 1792) | SPX |  |  | 1 |  |
|  | *Paromalus parallelepipedus* (Herbst, 1792) | SPX |  |  | 1 |  |
|  | *Platysoma compressum* (Herbst, 1783) | SPX |  | 1 |  |  |
|  | *Plegaderus caesus* (Herbst, 1792) | SPX |  |  |  | 1 |
| **Chrysomelidae** | *Oulema melanopus* (Linnaeus, 1758) | N-SPX |  | 1 |  |  |
|  | *Phyllotreta nigripes* (Fabricius, 1775) | N-SPX |  |  | 1 | 1 |
|  | *Psylliodes chrysocephalus* (Linnaeus, 1758) | N-SPX |  |  |  | 1 |
| **Lampyridae** | *Lamprohiza splendidula* (Linnaeus, 1767) | N-SPX |  |  | 2 | 4 |
| **Latridiidae** | *Cartodere nodifer* (Westwood, 1839) | N-SPX |  |  | 1 |  |
|  | *Corticaria longicornis* (Herbst, 1793) | SPX |  |  | 3 | 4 |
|  | *Corticaria serrata* (Paykull, 1798) | N-SPX |  |  |  | 1 |
|  | *Corticarina minuta* (Fabricius, 1792) | N-SPX |  |  |  | 2 |
|  | *Corticarina similata* (Gyllenhal, 1827) | N-SPX |  |  | 1 | 1 |
|  | *Cortinicara gibbosa* (Herbst, 1793) | N-SPX |  | 11 | 3 | 23 |
|  | *Enicmus atriceps* (Hansen, 1962) | SPX |  | 8 | 13 | 22 |
|  | *Enicmus brevicornis* (Mannerheim, 1844) | SPX |  |  |  | 3 |
|  | *Enicmus fungicola* (C.G. Thomson, 1868) | SPX |  | 1 | 10 | 7 |
|  | *Enicmus rugosus* (Herbst, 1793) | SPX |  | 92 | 131 | 286 |
|  | *Enicmus transversus* (Olivier, 1790) | N-SPX |  |  | 1 | 1 |
|  | *Latridius consimilis* (Mannerheim, 1844) | SPX |  |  | 1 |  |
|  | *Latridius hirtus* (Gyllenhal, 1827) | SPX |  | 6 | 9 | 15 |
|  | *Stephostethus alternans* (Mannerheim, 1844) | SPX |  | 5 | 4 | 4 |
|  | *Stephostethus angusticollis* (Gyllenhal, 1827) | N-SPX |  | 1 |  | 1 |
| **Leiodidae** | *Agathidium seminulum* (Linnaeus, 1758) | N-SPX |  | 29 | 18 | 60 |
|  | *Amphicyllis globiformis* (C. R. Sahlberg, 1833) | SPX |  |  |  | 2 |
|  | *Anisotoma humeralis* (Fabricius, 1792) | SPX |  |  |  | 2 |
|  | *Choleva cisteloides* (Frölich, 1799) | N-SPX |  | 1 |  | 7 |
|  | *Leiodes oblonga* (Erichson, 1845) | N-SPX |  | 1 |  | 4 |
|  | *Nemadus colonoides* (Kraatz, 1851) | SPX | NT | 1 |  |  |
| **Lucanidae** | *Platycerus caprea* (De Geer, 1774) | SPX |  |  | 1 | 1 |
|  | *Platycerus caraboides* (Linnaeus, 1758) | SPX |  |  | 2 | 1 |
|  | *Sinodendron cylindricum* (Linnaeus, 1758) | SPX |  | 7 | 4 | 8 |
| **Lymexylonidae** | *Hylecoetus dermestoides* (Linnaeus, 1761) | SPX |  | 6 | 1 | 3 |
| **Melandryidae** | *Abdera flexuosa* (Paykull, 1799) | SPX | NT |  | 2 | 9 |
|  | *Anisoxya fuscula* (Illiger, 1798) | SPX | NT |  |  | 1 |
|  | *Melandrya caraboides* (Linnaeus, 1760) | SPX | EN | 3 | 2 | 5 |
|  | *Orchesia fasciata* (Illiger, 1798) | SPX |  | 1 | 1 |  |
|  | *Orchesia micans* (Panzer, 1795) | SPX |  |  | 3 | 1 |
|  | *Orchesia undulata* (Kraatz, 1853) | SPX |  |  | 1 |  |
|  | *Phloiotrya rufipes* (Gyllenhal, 1810) | SPX |  | 1 | 5 | 9 |
|  | *Serropalpus barbatus* (Schaller, 1783) | SPX | NT |  |  | 2 |
| **Melyridae** | *Aplocnemus nigricornis* (Fabricius, 1792) | SPX |  |  | 1 | 1 |
|  | *Dasytes aeratus* (Stephens, 1829) | SPX |  |  | 1 |  |
|  | *Dasytes niger* (Linnaeus, 1761) | SPX |  |  | 3 | 4 |
|  | *Dasytes plumbeus* (O.F. Müller, 1776) | SPX |  | 643 | 415 | 1362 |
|  | *Hypebaeus flavipes* (Fabricius, 1787) | SPX |  |  |  | 1 |
| **Monotomidae** | *Rhizophagus bipustulatus* (Fabricius, 1792) | SPX |  | 51 | 110 | 39 |
|  | *Rhizophagus dispar* (Paykull, 1800) | SPX |  | 2 | 1 |  |
|  | *Rhizophagus fenestralis* (Linnaeus, 1758) | SPX |  | 1 | 1 | 3 |
|  | *Rhizophagus nitidulus* (Fabricius, 1798) | SPX | NT | 1 |  |  |
| **Mordelidae** | *Mordellistena neuwaldeggiana* (Panzer, 1796) | SPX |  | 1 | 17 | 8 |
|  | *Mordellistena variegata* (Fabricius, 1798) | SPX |  |  | 1 |  |
|  | *Mordellochroa abdominalis* (Fabricius, 1775) | SPX |  | 2 |  | 2 |
|  | *Tomoxia bucephala* (Costa, 1854) | SPX |  | 18 |  | 72 |
| **Mycetophagidae** | *Litargus connexus* (Geoffroy in Fourcroy, 1785) | SPX |  | 1 | 5 | 5 |
|  | *Mycetophagus atomarius* (Fabricius, 1787) | SPX |  | 1 | 1 | 4 |
|  | *Mycetophagus fulvicollis* (Fabricius, 1792) | SPX | VU |  | 2 | 3 |
|  | *Mycetophagus multipunctatus* (Fabricius, 1792) | SPX | NT |  | 1 |  |
|  | *Mycetophagus quadriguttatus* (P.W. J. Müller, 1821) | SPX |  |  | 2 |  |
|  | *Mycetophagus quadripustulatus* (Linnaeus, 1761) | SPX |  | 2 | 2 | 3 |
| **Nitidulidae** | *Brassicogethes aeneus* (Fabricius, 1775) | N-SPX |  | 85 | 84 | 122 |
|  | *Brassicogethes subaeneus* (Sturm, 1845) | N-SPX |  | 1 |  | 3 |
|  | *Cryptarcha strigata* (Fabricius, 1787) | SPX |  | 1 | 1 |  |
|  | *Cychramus variegatus* (Herbst, 1792) | SPX |  | 1 |  |  |
|  | *Epuraea aestiva* (Linnaeus, 1758) | N-SPX |  | 1 | 1 | 1 |
|  | *Epuraea longula* (Erichson, 1845) | SPX |  |  |  | 1 |
|  | *Epuraea melanocephala* (Marsham, 1802) | N-SPX |  |  | 2 | 3 |
|  | *Epuraea melina* (Erichson, 1843) | N-SPX |  | 1 |  |  |
|  | *Epuraea pallescens* (Stephens, 1835) | SPX |  | 2 |  |  |
|  | *Epuraea unicolor* (Olivier, 1790) | N-SPX |  | 3 |  | 3 |
|  | *Glischrochilus quadrisignatus* (Say, 1835) | N-SPX |  |  | 1 |  |
|  | *Glischrochilus hortensis* (Fourcroy, 1775) | N-SPX |  |  | 1 |  |
|  | *Glischrochilus quadriguttatus* (Fabricius, 1776) | SPX |  | 1 | 1 | 1 |
|  | *Glischrochilus quadripunctatus* (Linnaeus, 1758) | SPX |  |  | 1 |  |
|  | *Ipidia binotata* (Reitter, 1875) | SPX | NT | 2 |  | 1 |
|  | *Lamiogethes brunnicornis* (Sturm, 1845) | N-SPX |  | 49 | 5 | 25 |
|  | *Lamiogethes difficilis* (Heer, 1841) | N-SPX |  |  |  | 1 |
|  | *Lamiogethes morosus* (Erichson, 1845) | N-SPX |  |  |  | 1 |
|  | *Lamiogethes pedicularius* (Gyllenhal, 1808) | N-SPX |  | 3 |  | 6 |
|  | *Lamiogethes sulcatus* (C. Bri. de Barneville, 1863) | N-SPX |  |  | 1 | 1 |
|  | *Meligethes denticulatus* (Heer, 1841) | N-SPX |  |  |  | 4 |
|  | *Sagittogethes ovatus* (Sturm, 1845) | N-SPX |  | 2 |  |  |
|  | *Soronia grisea* (Linnaeus, 1758) | N-SPX |  | 6 | 46 | 20 |
|  | *Stachygethes ruficornis* (Marsham, 1802) | N-SPX |  | 1 |  |  |
|  | *Thalycra fervida* (Olivier, 1790) | N-SPX |  |  |  | 1 |
| **Oedemeridae** | *Ischnomera cyanea* (Fabricius, 1792) | SPX |  |  | 1 | 1 |
|  | *Oedemera podagrariae* (Linnaeus, 1767) | N-SPX |  |  | 1 | 1 |
| **Phalacridae** | *Olibrus corticalis* (Panzer, 1797) | N-SPX |  | 1 |  | 2 |
| **Ptiliidae** | *Acrotrichis intermedia* (Gillmeister, 1845) | N-SPX |  | 1 |  | 2 |
|  | *Ptenidium formicetorum* (Kraatz, 1851) | N-SPX |  |  |  | 1 |
| **Ptinidae** | *Dorcatoma minor* (Zahradník, 1993) | SPX |  |  |  | 1 |
|  | *Dryophilus pusillus* (Gyllenhal, 1808) | SPX |  |  | 1 |  |
|  | *Ernobius abietis* (Fabricius, 1792) | SPX |  | 2 | 3 | 2 |
|  | *Hadrobregmus pertinax* (Linnaeus, 1758) | SPX |  |  | 2 |  |
|  | *Hemicoelus costatus* (Aragona, 1830) | SPX |  | 13 | 24 | 10 |
|  | *Microbregma emarginatum* (Duftschmid, 1825) | SPX |  | 1 | 1 |  |
|  | *Priobium carpini* (Herbst, 1793) | SPX |  |  |  | 1 |
|  | *Pseudoptilinus fissicollis* (Saulcy & Weise, 1877) | SPX |  | 1 |  |  |
|  | *Ptilinus pectinicornis* (Linnaeus, 1758) | SPX |  | 13 | 36 | 16 |
|  | *Ptinomorphus imperialis* (Linnaeus, 1767) | SPX |  | 4 | 8 | 2 |
|  | *Ptinus subpillosus* (Sturm, 1837) | N-SPX |  | 3 |  | 2 |
|  | *Xestobium plumbeum* (Illiger, 1801) | SPX |  | 3 | 10 | 8 |
| **Pyrochroidae** | *Pyrochroa coccinea* (Linnaeus, 1761) | SPX |  | 1 | 1 | 4 |
|  | *Schizotus pectinicornis* (Linnaeus, 1758) | SPX |  | 2 |  | 7 |
| **Salpingidae** | *Salpingus planirostris* (Fabricius, 1787) | SPX |  |  | 2 | 2 |
|  | *Salpingus ruficollis* (Linnaeus, 1761) | SPX |  | 4 | 1 |  |
|  | *Vincenzellus ruficollis* (Panzer, 1794) | SPX |  | 34 | 121 | 29 |
| **Scarabaeidae** | *Acrossus depressus* (Kugelann, 1792) | N-SPX |  |  | 2 |  |
|  | *Agrilinus ater* (DeGeer, 1774) | N-SPX |  |  |  | 1 |
|  | *Melinopterus prodromus* (Brahm, 1790) | N-SPX |  | 1 | 1 |  |
|  | *Cetonia aurata* (Linnaeus, 1758) | SPX |  | 2 | 1 |  |
|  | *Limarus maculatus* (Sturm, 1800) | N-SPX |  | 1 |  |  |
|  | *Planolinus fasciatus* (Olivier, 1789) | N-SPX |  |  | 1 |  |
|  | *Protaetia cuprea* (Fabricius, 1775) | N-SPX |  | 1 | 1 |  |
|  | *Serica brunnea* (Linnaeus, 1758) | N-SPX |  |  | 4 |  |
|  | *Valgus hemipterus* (Linnaeus, 1758) | SPX |  | 1 |  |  |
|  | *Volinus sticticus* (Panzer, 1798) | N-SPX |  | 7 | 5 | 15 |
| **Scraptiidae** | *Anaspis flava* (Linnaeus, 1758) | SPX |  | 1 |  |  |
|  | *Anaspis marginicollis* (Lindberg, 1925) | SPX | VU | 2 | 3 | 1 |
|  | *Anaspis ruficollis* (Fabricius, 1792) | SPX |  | 1 | 2 | 1 |
|  | *Anaspis rufilabris* (Gyllenhal, 1827) | SPX |  | 23 | 23 | 22 |
| **Silphidae** | *Nicrophorus humator* (Gleditsch, 1767) | N-SPX |  | 2 |  |  |
|  | *Necrophagus vespiloides* (Herbst, 1784) | N-SPX |  | 2 |  |  |
|  | *Nicrophorus vespillo* (Linnaeus, 1758) | N-SPX |  | 1 | 4 | 6 |
|  | *Oiceoptoma thoracicum* (Linnaeus, 1758) | N-SPX |  |  |  | 1 |
| **Silvanidae** | *Silvanus unidentatus* (Olivier, 1790) | SPX |  |  | 1 |  |
|  | *Uleiota planata* (Linnaeus, 1761) | SPX |  | 1 | 2 | 6 |
| **Sphindidae** | *Aspidiphorus orbiculatus* (Gyllenhal, 1808) | SPX |  |  | 1 | 3 |
| **Tenebrionidae** | *Allecula morio* (Fabricius, 1787) | SPX | NT | 1 |  | 3 |
|  | *Bolitophagus reticulatus* (Linnaeus, 1767) | SPX |  |  |  | 1 |
|  | *Corticeus unicolor* (Piller & Mitterpacher, 1783) | SPX | NT | 2 | 6 | 12 |
|  | *Diaperis boleti* (Linnaeus, 1758) | SPX |  | 1 |  |  |
|  | *Mycetochara maura* (Fabricius, 1792) | SPX | NT | 1 | 1 | 8 |
|  | *Uloma culinaris* (Linnaeus, 1758) | SPX | NT |  | 1 | 2 |
| **Throscidae** | *Aulonothroscus brevicollis* (Bonvouloir, 1859) | N-SPX |  | 11 | 6 | 12 |
|  | *Trixagus carinifrons* (Bonvouloir, 1859) | N-SPX |  | 2 | 3 | 2 |
|  | *Trixagus dermestoides* (Linnaeus, 1766) | N-SPX |  | 66 | 164 | 51 |
|  | *Trixagus meybohmi* (Leseigneur, 2005) | N-SPX |  | 3 | 6 | 2 |
| **Trogossitidae** | *Nemozoma elongatum* (Linnaeus, 1761) | SPX |  | 5 | 23 | 19 |
| **Zopheridae** | *Bitoma crenata* (Fabricius, 1775) | SPX |  | 2 |  | 5 |
|  | *Colydium elongatum* (Fabricius, 1787) | SPX | NT | 1 | 1 | 1 |
